# Integrating services improve the return-to-work process for people on sick leave with stress-related disorders: results from a randomized trial (n=666)

**DOI:** 10.1192/j.eurpsy.2022.807

**Published:** 2022-09-01

**Authors:** A. Hoff

**Affiliations:** Capital Region Psychiatry, CORE - Copenhagen Research center for Mental Health, Capital Region Mental Health Services, KØBENHAVN N, Denmark

**Keywords:** vocational rehabilitation, Stress, integrated care, Exhaustion

## Abstract

**Introduction:**

Stress-related disorders are common and associated withsuffering and a large sociatal burden. While treatment appears to be able to reduce symptoms, evidence of interventions to improve work outcomes is inconsistent. Lack of integration different service domains has been suspected to be a barrier in return-to-work (RTW) processes.

**Objectives:**

We aimed to test the effectiveness of intergrating vocational rehabilitation and mental health care.

**Methods:**

We randomized participants on sick leave to I) service as usual (SAU), I) improved mental health care (MHC) or III) integrated interventions (INT). Primary outcome was RTW-rates measured at 12 months. Secondary outcome were proportion in work at 12 months, RTW-rates measured at 6 months, and symptom levels at 6 months.

**Results:**

We randomized 666 participants. Regarding primary outcome, the SAU group was superior to both MHC and INT. Furthermore, SAU was also superior to INT and MHC on almost all other work-related outcomes. INT and MHC did not show differences on any work-related outcome. On several symptom scales, MHC was observed with lower scores than SAU, whilst INT did not differ from the two other groups.

**Conclusions:**

Both the integrated intervention (INT) and the (non-integrated) mental health care (MHC) intervention lowered return-to-work rates compared with service as usual (SAU), and thereby yielded worse outcomes. However, the MHC group intervention showed a tendency towards having lower symptom levels compared with those in the SAU group; accordingly, the SAU group is not unequivocally superior. INT and MHC showed no general differences.

**Disclosure:**

No significant relationships.

